# Hydration behavior of D-calcium pantothenate (vitamin B5) in the presence of sugar-based deep eutectic solvents at different temperatures: experimental and theoretical study

**DOI:** 10.1038/s41598-024-75905-0

**Published:** 2024-10-22

**Authors:** Nadia Beladi, Fariba Ghaffari, Behrang Golmohammadi, Hemayat Shekaari

**Affiliations:** Department of Physical Chemistry, University of Tabriz, Tabriz, Iran

**Keywords:** Deep eutectic solvents, D-calcium pantothenate, Density, Speed of sound, Redlich-Meyer equation, Physical chemistry, Computational chemistry

## Abstract

Considerable efforts have been devoted in recent years to enhancing the efficacy medicinal substance, leading to the discovery of innovative drug formulations and delivery techniques. The successful design of these processes necessitates a profound understanding at the molecular level of how these substances interact with biological membranes. Thorough thermodynamic investigations provide invaluable insights into these interactions and aid in selecting suitable compounds for pharmaceutical production. This study aims to determine the density and speed of sound for D-calcium pantothenate in mixtures of water and deep eutectic solvents (DESs), specifically choline chloride/sucrose, choline chloride/ glucose, and choline chloride/ fructose (with 2:1 molar ratio) over a temperature range of 288.15 K to 318.15 K under atmospheric pressure. In order to predict the behavior of molecules, COSMO model (the Conductor-Like Screening Model) offer complementary strengths in quantum chemistry. This approach allows for calculating solvation free energies, making it ideal for predicting properties like solubility, where understanding solvent-solute interactions is crucial. By correlating the measured parameters using standard relationships, important partial molar parameters such as apparent molar volumes and apparent molar isentropic compressibility are calculated. Additionally, apparent molar isobaric expansion, and Hepler’s constant are derived from the density and speed of sound data. The experimental apparent molar volumes, and apparent molar isentropic compressibility data is fitted to the Redlich-Meyer equation to obtain significant quantities such as standard partial molar volume, and partial molar isentropic compression. The comprehensive thermodynamic analysis of this studied system holds immense significance for advancements in the pharmaceutical industry.

## Introduction

In cellular and organ functioning and development, vitamins play a crucial role as organic compounds. They are actively involved in enzymic processes and genetic regulation^1^. Understanding the physicochemical interactions between vitamins and key biomolecules such as amino acids, proteins, carbohydrates, and lipids is vital for comprehending the pharmacodynamics and pharmacokinetics of these compounds. Furthermore, this knowledge aids in the development of drug formulations within the pharmaceutical industry^2,3^. To gain deeper insights, researchers conduct comprehensive investigations into the thermophysical properties of vitamins in both water and aqueous media containing essential biomolecules. These studies aim to elucidate the molecular interactions between co-solutes and the hydrophilic and hydrophobic moieties of vitamins. Additionally, they seek to identify the conformational stability of biomolecules within biological systems^4,5^. Such research contributes to a better understanding of the behavior and interactions of vitamins with biomolecules, further advancing our knowledge of their roles in biological processes and pharmaceutical applications.

D-calcium pantothenate, also known as vitamin B5, plays a crucial role in various biochemical processes within the human body. This vitamin is a water-soluble compound and an integral component of coenzyme A (CoA)^6,7^. CoA is involved in vital metabolic reactions, including fatty acid synthesis, energy production, and acetylcholine synthesis. In recent years, the combination of vitamins with deep eutectic solvents has garnered considerable attention from researchers and scientists. This unique synergy has shown promising potential in diverse fields, ranging from pharmaceuticals to green chemistry^8,9^.

DESs are an emerging class of mixtures characterized by significant depressions in melting points compared to those of the neat constituent components. DESs formed through the combination of hydrogen bond donors (HBD) and acceptors (HBA), typically consisting of quaternary ammonium salts and hydrogen bond donors such as alcohols, carboxylic acids, and sugars. They exhibit unique physicochemical properties, including low melting points, non-volatility, and high thermal and chemical stability, making them attractive alternatives to conventional organic solvents in various applications^10,11^. These solvents have gained significant attention in recent years due to their environmentally friendly nature, biodegradability, and potential for sustainable processes^12–14^. Their tunable properties and ability to dissolve a wide range of compounds have led to their utilization in diverse fields, including catalysis, extraction, electrochemistry, and separation processes. Additionally, their biocompatibility and low toxicity open up possibilities for applications in biotechnology and pharmaceutical industries. The knowledge of volumetric and acoustic of D-calcium pantothenate with DESs is valuable in elucidating the assorted interactions prevailing in their solutions and for improving the design of various processes^15^.

Physicochemical and thermodynamic investigations are essential tools for understanding the complex nature and various types of molecular interactions in mixtures^16,17^. Thermodynamic properties, such as volume and compressibility, play a crucial role in elucidating the ionic, hydrophilic, and hydrophobic interactions in different solution media. These properties provide valuable insights into the interactions between solute and solvent molecules in the solution phase^18,19^. Accordingly, the Conductor-Like Screening Model provides a detailed picture of solute-solvent interactions; By employing this model, scientists have been able to glean significant information about the interplay between some ionic liquids and their environment, leading to more accurate predictions and improved their applications^20^. However, there is limited knowledge about the contributions of structurally similar DESs that influence the interactions of D-calcium pantothenate and its surroundings concerning temperature. Therefore, in this study, we measured and reported the densities (*d*) and speeds of sound (*u*) of D-calcium pantothenate (a derivative of vitamin B5) in water and DESs composed of choline chloride as HBA and sucrose, fructose and glucose as HBD (ChCl/S, ChCl/G, and ChCl/F) aqueous solutions at temperatures ranging from 288.15 K to 318.15 K with a 10 K interval as a function of concentration.

The data obtained from these measurements were then used to calculate various derived thermodynamic parameters, including the apparent molar volume, ${V_\varphi }$, standard partial molar volume, $V_{\varphi }^{0}$, apparent molar isentropic compression, ${\kappa _\varphi }$, and partial isentropic compression, $\kappa _{\varphi }^{0}$. These derived thermophysical parameters offer valuable insights into the performance and behavior of solvents during drug manufacturing processes. Understanding the interactions between D-calcium pantothenate and DESs can have significant implications for predicting and optimizing solvent behavior, ultimately contributing to more efficient drug manufacturing processes.

## Materials and methods

### Chemicals

The detailed descriptions of the used chemicals are specified in Table 1. To preparation of solutions double distilled deionized water with a conductivity of 0.055 µS/cm was applied at 298.15 K. Sucrose, glucose, and fructose were dried in vacuum over P_2_O_5_ at room temperature for at least 72 h.Other reagents were used without further purifications.

**Table 1:** Descriptions of the used chemicals.

Material	Provenance	CAS no.	Purity (mass fraction)	Purification method	^a^Water content (10^−6^.ppm)
Choline chloride	Daejung	67-48-1	> 0.99	Dried in vacuum over P_2_O_5_	0.0008
Glucose	Merck	50-99-7	> 0.995	Dried in vacuum over P_2_O_5_	0.0007
Fructose	Merck	57-48-7	≥ 0.99	Dried in vacuum over P_2_O_5_	0.0007
Sucrose	Merck	57-50-1	≥ 0.95	Dried in vacuum over P_2_O_5_	0.0007
D-calcium pantothenate	Daana Pharm. Co. (Tabriz Iran)	137-08-6	≥ 0.98	-	0.0002
Water	Deionized by distillation	-	> 0.995	Double distillation < 1 µS cm^−1^	-

^*a*^determined by Karl-Fischer method.

### Preparation of DESs

The preparation of all DESs was carried out using an electronic balance with a precision of ± 10^−7^ kg. The uncertainty in the DES composition, represented by the mole ratio of its constituent ingredients, was found to be within 4 × 10^−3^. In this research, three type of DESs were prepared using choline chloride as the hydrogen bond acceptor (HBA) and sucrose, glucose, and fructose as the hydrogen bond donor (HBD) in molar ratios of 2:1. The eutectic mixtures were prepared by stirring the two components at 60–80 ºC until a homogeneous transparent liquid was formed^21^. Subsequently, the mixture was left to cool down to room temperature naturally for further utilization^22^. The water content of the utilized DESs was determined through Karl-Fischer titration.

### Apparatus and procedure

For weighing the solutions, an analytical balance (AND, GF202, Japan) with an uncertainty of 10^−7^ kg was employed. These solutions were then carefully placed inside glass vials, which were securely sealed with parafilm to prevent any potential contamination. The density (*d*) and speed of sound (*u*) of the prepared solutions were measured using a vibrating tube densimeter (Anton Paar, DSA 5000 densimeter and speed of sound analyzer). To calibrate the apparatus, dry air at atmospheric pressure and degassed and double-distilled deionized water were utilized. The temperature during the experiments was maintained with a high level of precision, within an uncertainty of 10^−3^ K.

The measurements of density and speed of sound had uncertainties of 0.15 kg.m^−3^ and 0.5 m.s^−1^, respectively. These meticulous measurements and calibrations ensure the accuracy and reliability of the obtained data, allowing for comprehensive and precise analysis of the properties of the prepared solutions.

### Theoretical principles

In this study, we employed density functional theory (DFT) with the Dmol3 program to perform geometry optimizations of sucrose, fructose, glucose and D-calcium pantothenate. The Generalized Gradient Approximation (GGA) with the Vosko-Wilk-Nusair (VWN) functional supplemented by the BP functional (GGA-VWN-BP) was used. This approach replaces the local correlation component of the VWN functional, aligning with established COSMO results in the literature^20,23,24^.

## Results and discussions

### Volumetric properties

The experimental densities of D-calcium pantothenate in water and aqueous solutions of DESs (ChCl/S, ChCl/G, and ChCl/F) with different concentrations (0.1, 0.2, and 0.3 mol·kg^−1^) were investigated at various temperatures (288.15, 298.15, 308.15, and 318.15 K) and are presented in Table 2. In the studied concentration range of DESs, DESs may be converted into their initial materials. Therefore, instead of DES, their mixtures (ChCl/S, ChCl/G, and ChCl/F) are used.

**Table 2:** Density (*d*) and speed of sound (*u*) of D-calcium pantothenate in the aqueous solutions of mixtures of ChCl/S, ChCl/G, or ChCl/F at different temperatures and pressure (P = 86.6 kPa)^*a*^.

*m*_*1*_^*b*^/ mol∙kg^−1^	*T/*K = 288.15		*T/*K = 298.15		*T/*K = 308.15		*T/*K = 318.15	
	10^−3^*d*/ kg·m^−3^	u/ m·s^−1^	10^−3^*d*/ kg·m^−3^	u/ m·s^−1^	10^−3^*d*/ kg·m^−3^	u/ m·s^−1^	10^−3^*d*/ kg·m^−3^	u/ m·s^−1^
( ChCl/S molar ratio of 2.000:1.000) + D-calcium pantothenate + water								
^*c*^*m*_*2*_ = 0.100								
0.0000	1.00303	1476.93	1.00303	1506.99	1.00050	1528.98	1.00050	1544.24
0.1603	1.01073	1482.98	1.00732	1511.56	1.00449	1532.55	1.00449	1547.01
0.3184	1.01495	1490.07	1.01152	1516.54	1.00851	1536.98	1.00851	1550.55
0.4885	1.01960	1497.09	1.01616	1522.91	1.01304	1542.39	1.01304	1555.10
0.6504	1.02364	1505.49	1.02014	1530.42	1.01724	1547.95	1.01724	1560.19
0.8212	1.02806	1513.56	1.02464	1536.77	1.02142	1554.25	1.02142	1567.32
0.9828	1.03210	1522.68	1.02854	1543.8	1.02546	1561.06	1.02546	1574.81
*m*_*2*_ = 0.200								
0.000	1.01290	1488.42	1.00987	1517.15	1.00720	1537.69	1.00492	1551.90
0.1671	1.01683	1493.33	1.01365	1520.06	1.01070	1539.6	1.00814	1552.43
0.3223	1.02059	1499.06	1.01732	1524.59	1.01430	1543.02	1.01131	1555.25
0.4964	1.02454	1507.08	1.02132	1531.1	1.01837	1547.61	1.01512	1559.98
0.6576	1.02855	1513.64	1.02516	1537.6	1.02204	1553.85	1.01877	1565.53
0.8176	1.03235	1520.82	1.02896	1543.86	1.02594	1559.25	1.02211	1571.39
0.9801	1.03633	1527.93	1.03301	1550.21	1.03004	1564.25	1.02558	1576.86
*m*_*2*_ = 0.300								
0.000	1.01994	1501.62	1.01694	1527.99	1.01198	1549.24	1.00898	1563.57
0.1666	1.02210	1506.31	1.01904	1530.82	1.01380	1551.20	1.01075	1564.02
0.3217	1.02495	1511.37	1.02161	1534.89	1.01599	1554.64	1.01253	1567.32
0.5009	1.02822	1518.58	1.02450	1541.16	1.01955	1558.99	1.01600	1571.03
0.6566	1.03160	1524.13	1.02809	1545.23	1.02246	1563.37	1.01895	1575.20
0.8242	1.03532	1531.67	1.03184	1552.01	1.02654	1568.66	1.02244	1579.57
0.9852	1.03904	1538.96	1.03560	1557.45	1.03058	1573.15	1.02593	1584.88
( ChCl/G molar ratio of 2.000:1.000) + D-calcium pantothenate + water								
*m*_*2*_ = 0.100								
0.0000	1.00387	1474.65	1.00092	1504.64	0.99826	1526.75	0.99571	1542.45
0.1590	1.00850	1481.88	1.00519	1510.46	1.00237	1531.41	0.99966	1546.17
0.3206	1.01301	1489.15	1.00968	1516.62	1.00688	1536.02	1.00392	1549.99
0.4911	1.01803	1497.3	1.01459	1523.12	1.01158	1542.56	1.00823	1555.27
0.6425	1.02202	1504.83	1.01822	1529.76	1.01533	1548.25	1.01180	1560.49
0.8215	1.02703	1512.05	1.02360	1536.26	1.02040	1553.7	1.01620	1566.77
0.9943	1.03163	1519.81	1.02806	1541.93	1.02499	1559.15	1.02028	1572.9
*m*_*2*_ = 0.200								
0.0000	1.00755	1482.88	1.00476	1511.66	1.00221	1533.41	0.99988	1548.95
0.1615	1.01190	1489.03	1.00872	1516.76	1.00601	1537.3	1.00352	1552.13
0.2975	1.01549	1494.99	1.01227	1521.52	1.00960	1540.95	1.00673	1555.18
0.4892	1.02066	1503.21	1.01724	1528.23	1.01439	1546.74	1.01134	1560.11
0.6571	1.02478	1510.76	1.02139	1534.72	1.01826	1552.97	1.01540	1564.65
0.8210	1.02917	1518.36	1.02578	1541.4	1.02276	1557.79	1.01914	1569.93
0.9804	1.03329	1524.56	1.02997	1546.84	1.02701	1562.48	1.02272	1575.09
*m*_*2*_ = 0.300								
0.0000	1.01201	1492.12	1.00909	1521.73	1.00213	1538.48	0.99913	1558.72
0.1607	1.01594	1496.95	1.01258	1525.28	1.00574	1540.67	1.00269	1559.42
0.3276	1.01984	1503.52	1.01650	1529.2	1.00989	1543.6	1.00642	1561.31
0.4936	1.02404	1509.09	1.02041	1534.16	1.01397	1547.96	1.01042	1564.14
0.6522	1.02781	1515.98	1.02431	1539.41	1.01768	1552.57	1.01417	1568.37
0.8325	1.03214	1522.92	1.02866	1545.32	1.02235	1557.56	1.01825	1573.45
0.9887	1.03599	1529.11	1.03255	1551.58	1.02653	1563.3	1.02188	1578.77
( ChCl/F molar ratio of 2.000:1.000) + D-calcium pantothenate + water								
*m*_*2*_ = 0.100								
0.0000	1.00390	1474.69	1.00095	1504.68	0.99830	1526.83	0.99575	1542.53
0.1590	1.00855	1481.92	1.00524	1510.50	1.00241	1531.49	0.99973	1546.25
0.3206	1.01306	1489.19	1.00973	1516.66	1.00692	1536.10	1.00409	1550.07
0.4911	1.01808	1497.34	1.01464	1523.16	1.01162	1542.64	1.00839	1555.35
0.6425	1.02206	1504.87	1.01827	1529.80	1.01537	1548.33	1.01184	1560.57
0.8215	1.02708	1512.09	1.02365	1536.30	1.02043	1553.78	1.01624	1566.85
0.9943	1.03167	1519.85	1.02811	1541.97	1.02503	1559.23	1.02030	1572.98
*m*_*2*_ = 0.200								
0.0000	1.00760	1482.96	1.00481	1511.74	1.00226	1533.49	0.99993	1549.03
0.1615	1.01195	1489.11	1.00877	1516.84	1.00606	1537.38	1.00357	1552.21
0.2975	1.01555	1495.07	1.01233	1521.60	1.00966	1541.03	1.00678	1555.26
0.4892	1.02071	1503.29	1.01730	1528.31	1.01444	1546.82	1.01139	1560.19
0.6571	1.02483	1510.84	1.02144	1534.80	1.01831	1553.05	1.01545	1564.73
0.8210	1.02923	1518.44	1.02583	1541.48	1.02281	1557.87	1.01919	1570.01
0.9804	1.03334	1524.64	1.03002	1546.92	1.02706	1562.56	1.02277	1575.17
*m*_*2*_ = 0.300								
0.0000	1.01207	1492.24	1.00915	1521.85	1.00219	1538.60	0.99919	1558.84
0.1607	1.01600	1497.07	1.01264	1525.40	1.00580	1540.79	1.00275	1559.54
0.3276	1.01990	1503.64	1.01656	1529.32	1.00995	1543.72	1.00649	1561.43
0.4936	1.02410	1509.21	1.02048	1534.28	1.01403	1548.08	1.01048	1564.26
0.6522	1.02787	1516.10	1.02437	1539.53	1.01774	1552.69	1.01423	1568.49
0.8325	1.03220	1523.04	1.02872	1545.44	1.02242	1557.68	1.01831	1573.57
0.9887	1.03606	1529.23	1.03262	1551.7	1.02659	1563.42	1.02194	1578.89

^a^ The standard uncertainties (*u*) for density, speed of sound, temperature, pressure, molality of D-calcium pantothenate and DES are *u* (*d*) = 0.15 kg m^−3^; *u* (*u*) = 0.5 m s^−1^; u(*T*) = 0.01 K; u(*p*) = 0.5 kPa; *u*(m) = 0.002; *u*(DES) = 0.004, respectively with 0.68 level of confidence.

^b^*m*_1_ is the molal concentration of D-calcium pantothenate in the solution of DES + water.

^c^*m*_*2*_ is the molal concentration of DES in water.

Analysis of the data in Table 2 reveals a consistent trend where the densities increase monotonously with an increase in the concentration of the mixtures (ChCl/S, ChCl/G, and ChCl/F). Moreover, for a fixed D-calcium pantothenate concentration, the densities decrease as the temperature rises. This information indicates the influence of (ChCl/S, ChCl/G, and ChCl/F) concentration and temperature on the densities of the D-calcium pantothenate solutions, providing valuable insights into the behavior of these systems. By using the Eq. (1) apparent molar volumes, ${V_\varphi }$, could be calculated from the experimental density values^25,26^:

1 $${V_\varphi }=\frac{M}{d} - \left[ {\frac{{(d - {d_0})}}{{md{d_0}}}} \right]$$

**Figure 1:**
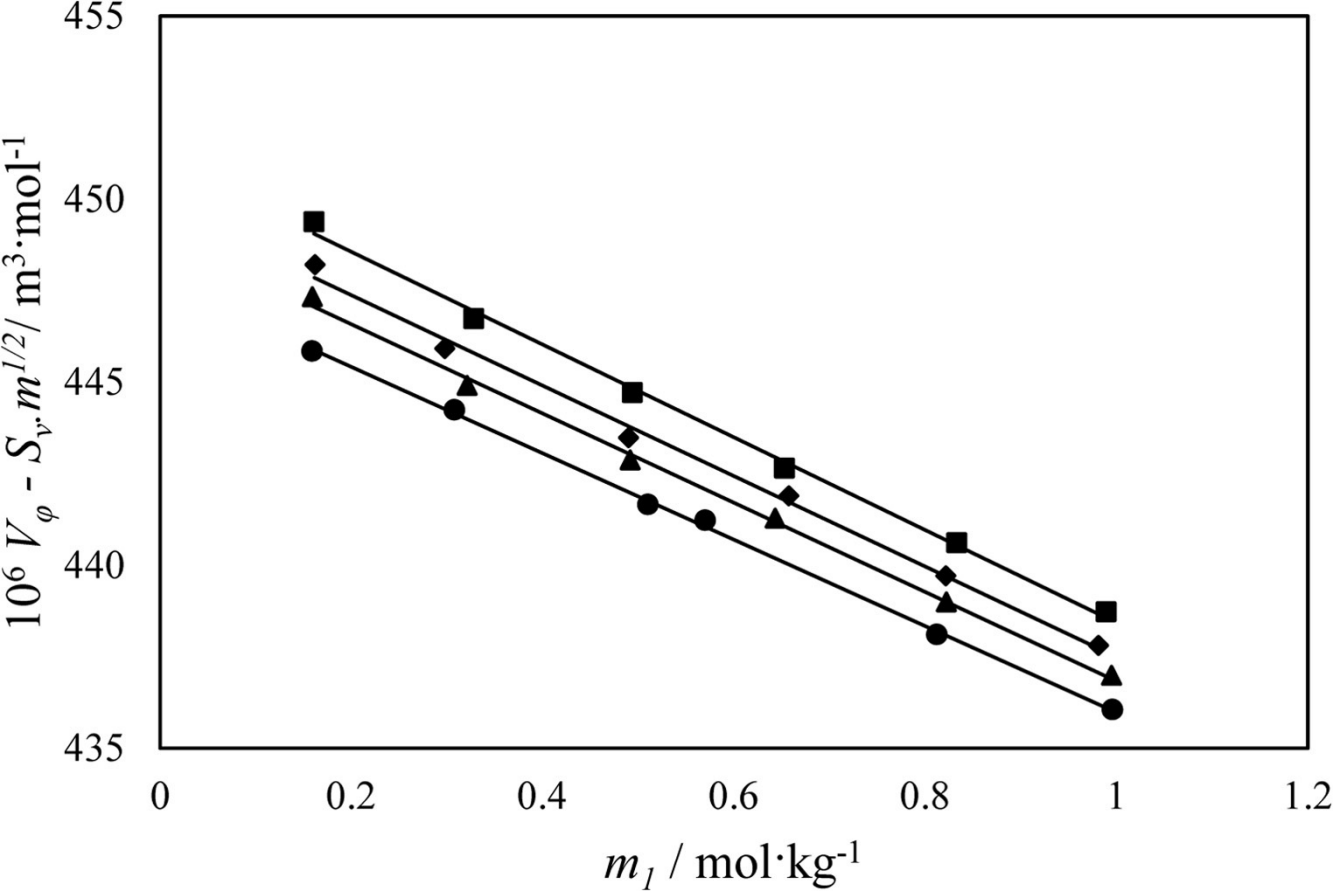
Apparent molar volume of D-calcium pantothenate, ${V_\varphi }$, versus its molality, *m*_1_, in aqueous ChCl/G solutions at *T* = 298.15 K: ●, binary; ▲, 0.1; ♦, 0.2; ■, 0.3 mol·kg^−1^.

In the investigated solutions, the variables “*m*” and “*M*” represent the molalities and molecular mass of D-calcium pantothenate, respectively. Likewise, “*d*_*0*_” and “*d*” correspond to the densities of the solvent and solutions, respectively. For the ternary systems, the solvent is considered to be a mixture of DES and water. The experimental values of apparent molar volume for D-calcium pantothenate in both pure water and aqueous solutions containing each mixture (ChCl/S, ChCl/G, and ChCl/F) with combined uncertainty of 0.06 × 10^−6^ m^3^·mol^−1^ at various temperatures are documented in Table 3. Furthermore, Fig. 1 displays the plot of apparent molar volumes for D-calcium pantothenate at a fixed molality of each mixture (ChCl/S, ChCl/G, and ChCl/F) (0.3 mol·kg^−1^) and at a temperature of 298.15 K. From the results, it is evident that the apparent molar volumes increase with the growing concentration of DES and temperature. This observation suggests a direct influence of ChCl/S, ChCl/G, or ChCl/F concentration on the apparent molar volume of D-calcium pantothenate in the studied solutions.

**Table 3:** The apparent molar volumes ($V_{\varphi }^{{}}$) and apparent molar isentropic compressibility ($k_{\varphi }$) of D-calcium pantothenate in aqueous solutions of mixtures of ChCl/S, ChCl/G, or ChCl/F at different temperatures and pressure (P = 86.6 kPa)^*a*^.

^*b*^*m*/ mol∙kg^−1^	*T* / K = 288.15		*T* / K = 298.15		*T* / K = 308.15		*T* / K = 318.15	
	*10*^*6*^$V_{\varphi }^{{}}$*/* m^3^·mol^−1^	*10*^*14*^$k_{\varphi }$*/* m^3^·mol^−1^·Pa^−1^	*10*^*6*^$V_{\varphi }^{{}}$*/* m^3^·mol^−1^	*10*^*14*^$k_{\varphi }$*/* m^3^·mol^−1^·Pa^−1^	*10*^*6*^$V_{\varphi }^{{}}$*/* m^3^·mol^−1^	*10*^*14*^$k_{\varphi }$*/* m^3^·mol^−1^·Pa^−1^	*10*^*6*^$V_{\varphi }^{{}}$*/* m^3^·mol^−1^	*10*^*14*^$k_{\varphi }$*/* m^3^·mol^−1^·Pa^−1^
( ChCl/S molar ratio of 2.000:1.000) + D-calcium pantothenate + water								
*m*_*2*_ = 0.100								
0.1588	444.365	16.467	446.582	16.600	449.635	16.764	452.110	16.930
0.3075	442.927	15.969	444.83	16.257	447.563	16.337	449.955	16.539
0.5098	440.848	15.636	442.579	15.782	445.064	15.889	448.030	16.157
0.5697	439.654	15.200	441.405	15.335	443.157	15.525	446.497	15.806
0.8122	437.910	14.847	439.468	15.035	441.601	15.187	445.165	15.365
0.9956	436.437	14.429	438.139	14.722	439.942	14.821	443.951	14.964
*m*_*2*_ = 0.200								
0.008	445.803	16.92	448.009	17.206	450.923	17.342	453.681	17.720
0.029	443.832	16.407	445.923	16.613	448.254	16.770	451.715	17.116
0.060	442.530	15.854	444.232	16.053	446.014	16.304	449.309	16.510
0.089	440.458	15.496	442.375	15.600	444.346	15.794	447.181	15.990
0.120	438.852	15.14	440.660	15.247	442.312	15.427	445.751	15.597
0.148	437.058	14.811	438.674	14.904	440.170	15.121	444.196	15.283
*m*_*2*_ = 0.300								
0.008	453.611	17.437	455.346	17.643	459.498	17.779	461.398	18.084
0.029	450.462	16.878	452.916	17.062	456.567	17.233	458.833	17.454
0.060	447.401	16.320	450.033	16.513	453.225	16.662	455.826	16.849
0.089	445.005	15.956	447.494	16.151	450.343	16.293	453.195	16.423
0.120	442.596	15.474	444.739	15.641	447.254	15.807	450.349	16.038
0.148	440.402	15.062	442.075	15.297	444.293	15.452	447.604	15.635
( ChCl/G molar ratio of 2.000:1.000) + D-calcium pantothenate + water								
*m*_*2*_ = 0.100								
0.1590	443.81	15.966	447.285	16.215	449.419	16.407	451.619	16.586
0.3206	442.035	15.593	444.911	15.794	446.766	15.955	449.276	16.229
0.4911	440.204	15.255	442.716	15.441	444.449	15.580	447.328	15.877
0.6425	438.598	14.980	440.904	15.167	442.607	15.293	445.832	15.576
0.8215	436.715	14.673	438.869	14.874	440.594	14.989	444.243	15.229
0.9943	434.906	14.391	436.98	14.614	438.77	14.721	442.838	14.900
*m*_*2*_ = 0.200								
0.1615	444.512	16.339	448.094	16.456	450.036	16.628	452.457	16.728
0.2975	443.085	15.949	446.101	16.103	447.978	16.252	450.363	16.425
0.4892	441.089	15.505	443.612	15.675	445.434	15.819	447.981	16.040
0.6571	439.347	15.169	441.588	15.335	443.381	15.489	446.174	15.723
0.821	437.652	14.869	439.7	15.022	441.475	15.192	444.565	15.425
0.9804	436.005	14.597	437.921	14.729	439.686	14.922	443.104	15.142
*m*_*2*_ = 0.300								
0.1607	445.347	16.738	449.341	16.953	451.412	17.239	453.245	17.455
0.3276	443.752	16.271	446.838	16.572	448.794	16.789	451.044	17.046
0.4936	442.066	15.887	444.638	16.196	446.507	16.378	449.076	16.644
0.6522	440.409	15.561	442.677	15.838	444.475	16.005	447.303	16.262
0.8325	438.489	15.218	440.55	15.432	442.277	15.595	445.367	15.829
0.9887	436.805	14.94	438.773	15.080	440.444	15.248	443.739	15.456
( ChCl/F molar ratio of 2.000:1.000) + D-calcium pantothenate + water								
*m*_*2*_ = 0.100								
0.159	443.731	15.958	447.204	16.207	449.402	16.404	451.327	16.560
0.3206	441.979	15.587	444.857	15.789	446.750	15.952	448.952	16.200
0.4911	440.164	15.251	442.676	15.437	444.433	15.578	447.057	15.853
0.6425	438.566	14.976	440.872	15.164	442.591	15.291	445.647	15.559
0.8215	436.690	14.671	438.840	14.872	440.579	14.987	444.189	15.223
0.9943	434.885	14.389	436.950	14.611	438.754	14.718	442.932	14.906
*m*_*2*_ = 0.200								
0.1615	444.49	16.336	448.072	16.452	450.014	16.625	452.435	16.724
0.2975	443.064	15.946	446.08	16.100	447.956	16.249	450.341	16.422
0.4892	441.067	15.502	443.591	15.672	445.412	15.816	447.959	16.037
0.6571	439.326	15.166	441.567	15.332	443.36	15.486	446.152	15.720
0.821	437.631	14.867	439.679	15.019	441.454	15.189	444.544	15.422
0.9804	435.985	14.594	437.901	14.727	439.665	14.919	443.082	15.139
*m*_*2*_ = 0.300								
0.1607	445.321	16.734	449.315	16.949	451.385	17.235	453.219	17.450
0.3276	443.726	16.266	446.812	16.568	448.768	16.784	451.018	17.042
0.4936	442.041	15.883	444.613	16.192	446.481	16.374	449.05	16.640
0.6522	440.384	15.557	442.651	15.834	444.449	16.001	447.277	16.258
0.8325	438.464	15.214	440.525	15.428	442.252	15.591	445.341	15.825
0.9887	436.780	14.936	438.748	15.076	440.419	15.244	443.713	15.452

^*a*^The standard uncertainties (*u*) for density, speed of sound, temperature, pressure, molality of D-calcium pantothenate and DES are *u* (*d*) = 0.15 kg m^−3^; *u* (u) = 0.5 m s^−1^; u(*T*) = 0.01 K; u(*p*) = 0.5 kPa; *u*(m) = 0.002; *u*(DES) = 0.004, respectively. The combined uncertainty for apparent molar volumes and apparent molar isentropic compressibility are *u*_*c*_ (${V}_{\varphi }$) = 0.06 $\times$ 10^–6^ m^3^·mol^−1^ and *u*_*c*_ ($k_{\varphi }$ ) = 0.02 × 10^–14^ m^3^mol Pa^−1^ (0.68 level of confidence), respectively.

^*b*^* m*_1_ is the molal concentration of D-calcium pantothenate in the solution of DES + water.

^*c*^* m*_*2*_ is the molal concentration of DES in water.

The following Redlich-Mayer equation was used to obtain the standard partial molar volume, ${V_\varphi }^{0}$^27^:

2 $$V_\varphi=V_\varphi^0+S_vm^{1/2}+b_vm$$

The ${V_\varphi }^{0}$parameter represents the standard partial molar volume, specifically the partial molar volume at infinite dilution. Empirical parameters *S*_*v*_ and *b*_*v*_ are also utilized in this context. Given that solute-solute interactions are negligible under infinite dilution conditions, the standard partial molar volumes offer valuable insights into solute-solvent interactions. In Table 4, we present the values of ${V_\varphi }^{0}$, *S*_*v*_ and *b*_*v*_, along with their corresponding standard deviations. Notably, all values, serving as indicators of solute-solvent interactions, exhibit positive values. Moreover, these values increase with higher concentrations of ChCl/S, ChCl/G, or ChCl/F and at elevated temperatures. This behavior can be attributed to the weaker electrostriction of water and the enhanced interactions between solute and solvent molecules. The larger values observed at higher temperatures likely correspond to the release of solvent molecules into the bulk. The ${V_\varphi }^{0}$values provide information about the solute–solvent interactions for D-calcium pantothenate in aqueous solutions of DESs as the following order: ChCl/S > ChCl/G > ChCl/F, respectively. This order interpreted that the interactions between D-calcium pantothenate as solute and solvent were strengthened with ChCl/S, ChCl/G, or ChCl/F. The ${V_\varphi }^{0}$ values for D-calcium pantothenate are very close for two ChCl/G, and ChCl/F. This behavior is maybe because of structure and size of studied sugars as HBD. In the case of ternary systems, the positive *b*_*v*_ values indicate the dominance of hydrophilic hydrogen-bonding interactions over hydrophobic interactions^28,29^. These findings shed light on the nature and strength of solute-solvent interactions, particularly in the presence of mixtures of ChCl/S, ChCl/G, or ChCl/F, offering crucial implications for understanding solution behavior and properties.

**Table 4:** Standard partial molar volumes (),adjustable parameters of Eq. 18 ($~{S_v}~$and ), transfer volume ($~{\Delta _{tr}}V_{\varphi }^{0}~$), and standard deviations () for D-calcium pantothenate in aqueous solutions of mixtures of ChCl/S, ChCl/G, or ChCl/F at different temperatures^*a*^.

T / K	$~V_{\varphi }^{0}~$⋅10^6^/ (m^3^·mol^−1^)	S_v_·10^6^ / (m^3^·mol^−2^·kg)	B_v_·10^6^ / (m^3^·mol^−2^·kg)	$~{\Delta _{tr}}V_{\varphi }^{0}~$·10^6^/ (m^3^·mol^−1^)	$\sigma (~V_{\varphi }^{0}~)$·10^6^/ (m^3^·mol^−1^)
( ChCl/S molar ratio of 2.000:1.000) + D-calcium pantothenate + water					
*m*_2_ = 0.100 mol·kg^−1^					
288.15	446.630	-2.341	-8.011	3.596	0.141
298.15	450.086	-6.319	-5.821	1.009	0.170
308.15	454.602	-10.395	-4.456	1.374	0.036
318.15	457.464	-13.100	-0.544	2.061	0.149
*m*_2_ = 0.200 mol·kg^−1^					
288.15	447.326	8.440	-15.916	4.292	0.136
298.15	450.140	6.086	-14.790	1.063	0.082
308.15	455.131	-3.486	-10.036	1.903	0.205
318.15	458.593	-5.150	-6.881	3.190	0.137
*m*_2_ = 0.300 mol·kg^−1^					
288.15	460.165	-13.367	-6.593	17.131	0.245
298.15	457.539	1.755	-17.464	8.462	0.366
308.15	462.891	-1.033	-17.836	9.663	0.553
318.15	463.911	1.016	-17.575	8.508	0.294
( ChCl/G molar ratio of 2.000:1.000) + D-calcium pantothenate + water					
*m*_2_ = 0.100 mol·kg^−1^					
288.15	445.793	-1.011	-9.936	2.759	0.219
298.15	451.344	-7.361	-7.065	2.267	0.385
308.15	454.714	-11.471	-4.532	1.486	0.177
318.15	456.844	-12.473	-1.577	1.441	0.292
*m*_2_ = 0.200 mol·kg^−1^					
288.15	446.274	-0.299	-10.173	3.240	0.176
298.15	451.989	-6.608	-7.675	2.912	0.183
308.15	454.19	-7.395	-7.326	0.962	0.084
318.15	457.672	-11.793	-2.95	2.269	0.432
*m*_2_ = 0.300 mol·kg^−1^					
288.15	446.308	2.439	-12.065	3.274	0.250
298.15	453.449	-7.198	-7.605	4.372	0.290
308.15	455.785	-7.855	-7.616	2.557	0.180
318.15	456.664	-5.505	-7.537	1.261	0.290
( ChCl/F molar ratio of 2.000:1.000) + D-calcium pantothenate + water					
*m*_2_ = 0.100 mol·kg^−1^					
288.15	445.636	-0.779	-10.032	2.602	0.221
298.15	451.157	-7.023	-7.246	2.080	0.385
308.15	454.697	-11.47	-4.532	1.469	0.293
318.15	457.064	-14.529	0.357	1.661	0.292
*m*_2_ = 0.200 mol·kg^−1^					
288.15	446.253	-0.299	-10.172	3.219	0.176
298.15	451.967	-6.608	-7.673	2.890	0.183
308.15	454.168	-7.395	-7.325	0.940	0.084
318.15	457.650	-11.792	-2.949	2.247	0.432
*m*_2_ = 0.300 mol·kg^−1^					
288.15	446.282	2.439	-12.064	3.248	0.193
298.15	453.422	-7.197	-7.604	4.345	0.068
308.15	455.758	-7.854	-7.615	2.530	0.177
318.15	456.636	-5.504	-7.536	1.233	0.272

^*a*^The standard uncertainties (*u*) for temperature, pressure, molality of D-calcium pantothenate and DES are u(*T*) = 0.01 K; u(*p*) = 0.5 kPa; *u*(m) = 0.002; *u*(DES) = 0.004, respectively. The combined uncertainty for apparent molar volumes is *u*_*c*_ ($\:{V}_{\phi\:}$) = 0.06$\:\times\:$10^−6^ m^3^·mol^−1^ with 0.68 level of confidence.

^*b*^*m*_*2*_ is the molal concentration of DES in water.

Temperature dependence of $V_{\varphi }^{0}$ values can be defined by following equation:

3 $${V_\varphi }^{0}=A+BT+C{T^2}$$

where *A*,* B* and *C* are empirical constants which are calculated by the least-square fitting of standard partial molar volume at studied temperatures^30,31^.

By differentiating the standard partial molar volume with respect to temperature at constant pressure, the standard apparent molar expansibilities$E_{\varphi }^{0}$, were computed. The obtained values are presented in Table 5. In the context of aqueous solutions of the studied drug with mixtures of ChCl/S, ChCl/G, or ChCl/F, the values of are consistently positive. This positive expansibility signifies a characteristic property of hydrophobic hydration in such solutions. The presence of hydrophobic groups in the drug molecules leads to an increased volume of the solution relative to pure water, resulting in a positive value for$E_{\varphi }^{0}$. Consequently, the aqueous mixtures of ChCl/S, ChCl/G, or ChCl/F solutions exhibit an enhanced expansion in volume compared to pure water due to this hydrophobic hydration effect.

**Table 5:** Standard apparent molar expansibility, $E_{\varphi }^{0}$, coefficient of thermal expansion, $\alpha$, Hepler’s constant expansion, ${({\partial ^2}V_{\varphi }^{0}/\partial {T^2})_P}$ of D-calcium pantothenate in water and in the aqueous solutions of mixtures of ChCl/S, ChCl/G, or ChCl/F at *T* = (288.15 to 318.15) K^*a*^.

T / K	$\:{(E}_{\phi\:}^{0})$/ m^3^·mol^−1^·K^−1^	10^3^$\:\alpha\:$/K^−1^	10^4^$\:{(\partial\:}^{2}{V}_{\phi\:}^{0}$/$\:\partial\:{T}^{2}$)_*P*_
( ChCl/S molar ratio of 2.000:1.000) + D-calcium pantothenate + water			
*m*_2_ = 0.100 mol·kg^−1^			
288.15	0.4387	1.2539	-0.0020
298.15	0.4587	1.4951	
308.15	0.4787	1.7799	
318.15	0.4987	2.0567	
*m*_2_ = 0.200 mol·kg^−1^			
288.15	0.3002	1.2625	0.0080
298.15	0.3802	1.5655	
308.15	0.4602	1.8660	
318.15	0.5402	2.1321	
*m*_2_ = 0.300 mol·kg^−1^			
288.15	-0.108	-2.337	0.0182
298.15	0.075	1.634	
308.15	0.257	5.553	
318.15	0.439	9.470	
( ChCl/F molar ratio of 2.000:1.000) + D-calcium pantothenate + water			
*m*_2_ = 0.100 mol·kg^−1^			
288.15	0.622	13.947	-0.0171
298.15	0.451	9.986	
308.15	0.28	6.151	
318.15	0.109	2.379	
*m*_2_ = 0.200 mol·kg^−1^			
288.15	0.531	11.908	-0.0112
298.15	0.42	9.287	
308.15	0.308	6.784	
318.15	0.197	4.294	
*m*_2_ = 0.300 mol·kg^−1^			
288.15	0.804	18.008	-0.0313
298.15	0.491	10.819	
308.15	0.177	3.894	
318.15	-0.136	-2.971	
( ChCl/F molar ratio of 2.000:1.000) + D-calcium pantothenate + water			
*m*_2_ = 0.100 mol·kg^−1^			
288.15	0.615	13.794	-0.0158
298.15	0.457	10.131	
308.15	0.299	6.585	
318.15	0.142	3.101	
*m*_2_ = 0.200 mol·kg^−1^			
288.15	0.531	11.907	-0.0112
298.15	0.420	9.287	
308.15	0.308	6.784	
318.15	0.196	4.293	
*m*_2_ = 0.300 mol·kg^−1^			
288.15	0.804	18.007	-0.0313
298.15	0.491	10.819	
308.15	0.177	3.893	
318.15	-0.136	-2.970	

^a^The standard uncertainties for molality, temperature, pressure, and prepared DESs were *u* (*m*) *=* 0.001 mol kg^−1^, *u* (*T*) *=* 0.2 K, *u* (*P*) *=* 0.5 kPa; *u*(DES) = 0.004, respectively with 0.68 level of confidence.

The values of $E_{\varphi }^{0}$ exhibit a positive trend, displaying an increase with both higher concentrations of mixtures of ChCl/S, ChCl/G, or ChCl/F and elevated temperatures. This observation suggests a notable sensitivity of the systems to temperature variations, indicating that molecular motilities are enhanced at higher temperatures^32,33^.

The thermal expansion coefficient, $\alpha$, was calculated by the values of the standard partial molar volume, using Eq. (4)^34^:

4 $$\alpha =\frac{{E_{\varphi }^{0}}}{{V_{\varphi }^{0}}}$$

The thermal expansion coefficients, denoted as $\alpha$, have been documented in Table 5 for the examined systems. This parameter serves as a crucial indicator to assess how the solutions respond to changes in temperature. The sign of the second derivative of the volume ($V_{\varphi }^{0}$) with respect to temperature allows us to obtain qualitative insights into the solute’s behavior as a structure maker or structure breaker in the solutions. The following expression^35^ is employed to achieve this analysis:

5 $${({\raise0.7ex\hbox{${\partial \,E_{\varphi }^{0}}$} \!\mathord{\left/ {\vphantom {{\partial \,E_{\varphi }^{0}} {\partial T}}}\right.\kern-0pt}\!\lower0.7ex\hbox{${\partial T}$}})_P}={({\raise0.7ex\hbox{${{\partial ^2}V_{\varphi }^{0}}$} \!\mathord{\left/ {\vphantom {{{\partial ^2}V_{\varphi }^{0}} {\partial {T^2}}}}\right.\kern-0pt}\!\lower0.7ex\hbox{${\partial {T^2}}$}})_\text{P}}=2 C$$

By analyzing the thermal expansion coefficients and the second derivative of volume with respect to temperature, we can gain valuable information regarding the role of the solute in influencing the solution’s structure and its responsiveness to temperature variations.

The ${({\partial ^2}{V_\varphi }^{0}/\partial {T^2})_p}$values of the investigated systems are presented in Table 5. According to previous research^36^, a negative value of ${({\partial ^2}{V_\varphi }^{0}/\partial {T^2})_p}$ suggests a structure-breaking solute, whereas a positive value or one closer to zero indicates a structure-making solute. Our observations reveal that the values of V for aqueous solutions of D-calcium pantothenate tend to be negative and closer to zero. However, as the concentration of mixtures of ChCl/S, ChCl/G, or ChCl/F increases, these values transition to positive values. This behavior implies that the drug, D-calcium pantothenate, predominantly acts as a structure maker in the aqueous mixtures of ChCl/S, ChCl/G, or ChCl/F solutions, influencing their structural characteristics. The results shed light on the solute’s impact on the solution’s structure and offer valuable insights for understanding the behavior of these systems.

The partial molar volume of transfer, ${\Delta _{tr}}\mathop V\nolimits_{\varphi }^{0}$, for D-calcium pantothenate from water to the aqueous mixtures of ChCl/S, ChCl/G, or ChCl/F solutions have been calculated as^37^:

6 $${\Delta _{tr}}\mathop V\nolimits_{\varphi }^{0} =\mathop V\nolimits_{\varphi }^{0} ({\text{DES+water}}) - \mathop V\nolimits_{\varphi }^{0} ({\text{water}})$$

The ${\Delta _{tr}}\mathop V\nolimits_{\varphi }^{0}$ values of the studied solutions are reported in Table 4. The values of $V_{\varphi }^{0}$ for binary (D-calcium pantothenate + water) was taken from reference^38^. These ${\Delta _{tr}}\mathop V\nolimits_{\varphi }^{0}$ values demonstrate a positive trend at all temperatures and exhibit an increase with the growing concentration of each mixture of ChCl/S, ChCl/G, or ChCl/F. The interactions between the solute, D-calcium pantothenate, and the co-solute, mixtures of ChCl/S, ChCl/G, or ChCl/F, in the ternary solutions (D-calcium pantothenate + (ChCl/S, ChCl/G, or ChCl/F) + water) encompass several possibilities: (i) ion-hydrophilic and hydrophilic-hydrophilic interactions between ions of D-calcium pantothenate and the polar groups of ChCl/S, ChCl/G, or ChCl/F; (ii) hydrophobic interactions between ions of D-calcium pantothenate and the non-polar groups of ChCl/S, ChCl/G, or ChCl/F; (iii) hydrophobic-hydrophobic interactions between the alkyl groups of the drug and the hydrophobic groups of ChCl/S, ChCl/G, or ChCl/F; and (iv) hydrophilic-hydrophobic interactions between the cation and anion components of D-calcium pantothenate and the hydrophilic groups of mixtures of ChCl/S, ChCl/G, or ChCl/F.

According to the co-sphere overlap model^39,40^, two types of interactions, namely, ion-hydrophilic and hydrophilic-hydrophilic interactions, result in positive effects on the ${\Delta _{tr}}\mathop V\nolimits_{\varphi }^{0}$ values, while the other types lead to negative transfer volumes. The presence of positive ${\Delta _{tr}}\mathop V\nolimits_{\varphi }^{0}$ values suggests that hydrophilic interactions between the COOH and CH_2_OH groups predominate over other types of interactions, and this trend becomes more pronounced with increasing concentrations of each mixtures of ChCl/S, ChCl/G, or ChCl/F^41,42^.

The findings indicate the significance of hydrophilic interactions in influencing the volumetric behavior of the solutions and provide valuable insights into the molecular interactions occurring in these systems.

### Ultrasonic properties

Experimentally measured speeds of sound, *u*, for D-calcium pantothenate in water and aqueous solutions of mixtures of ChCl/S, ChCl/G, or ChCl/F at different temperatures are listed in Table 2. The Laplace–Newton’s equation^43^ was used to obtain the isentropic compressibility value, ${\kappa _s}$ by following equation:

7 $$\kappa_s={\textstyle\frac1{du^2}}$$

The overall isentropic compressibility (${\kappa _s}$) of a system is composed of two components: ${\kappa _{s1}}$to solvent intrinsic and ${\kappa _{s2}}$ to solute intrinsic. The former is associated with the compression of the solvent, which can be either water or aqueous solutions of mixtures of ChCl/S, ChCl/G, or ChCl/F. The latter, ${\kappa _{s1}}$ (solute intrinsic), accounts for the compression layer of solute molecules influenced by the surrounding solvent molecules occupying the empty spaces around the solute. Notably, at low concentrations of D-calcium pantothenate, the dominant contribution to the overall ${\kappa _{s1}}$ value is attributed to ${\kappa _s}$ (solvent intrinsic)^44^. As the concentration of mixtures of ChCl/S, ChCl/G, or ChCl/F increases, lower values of ${\kappa _s}$ are observed. This behavior can be attributed to the disruption of the three-dimensional structure of water caused by the formation of hydrogen bonds around the D-calcium pantothenate molecules. Consequently, this phenomenon leads to a reduction in the compression of water molecules within the bulk solution. The interplay between solvent and solute molecules and their respective compressibility contributions influences the overall isentropic compressibility of the system. These findings highlight the complex nature of molecular interactions within the solution and their impact on its compressibility behavior.

**Figure 2:**
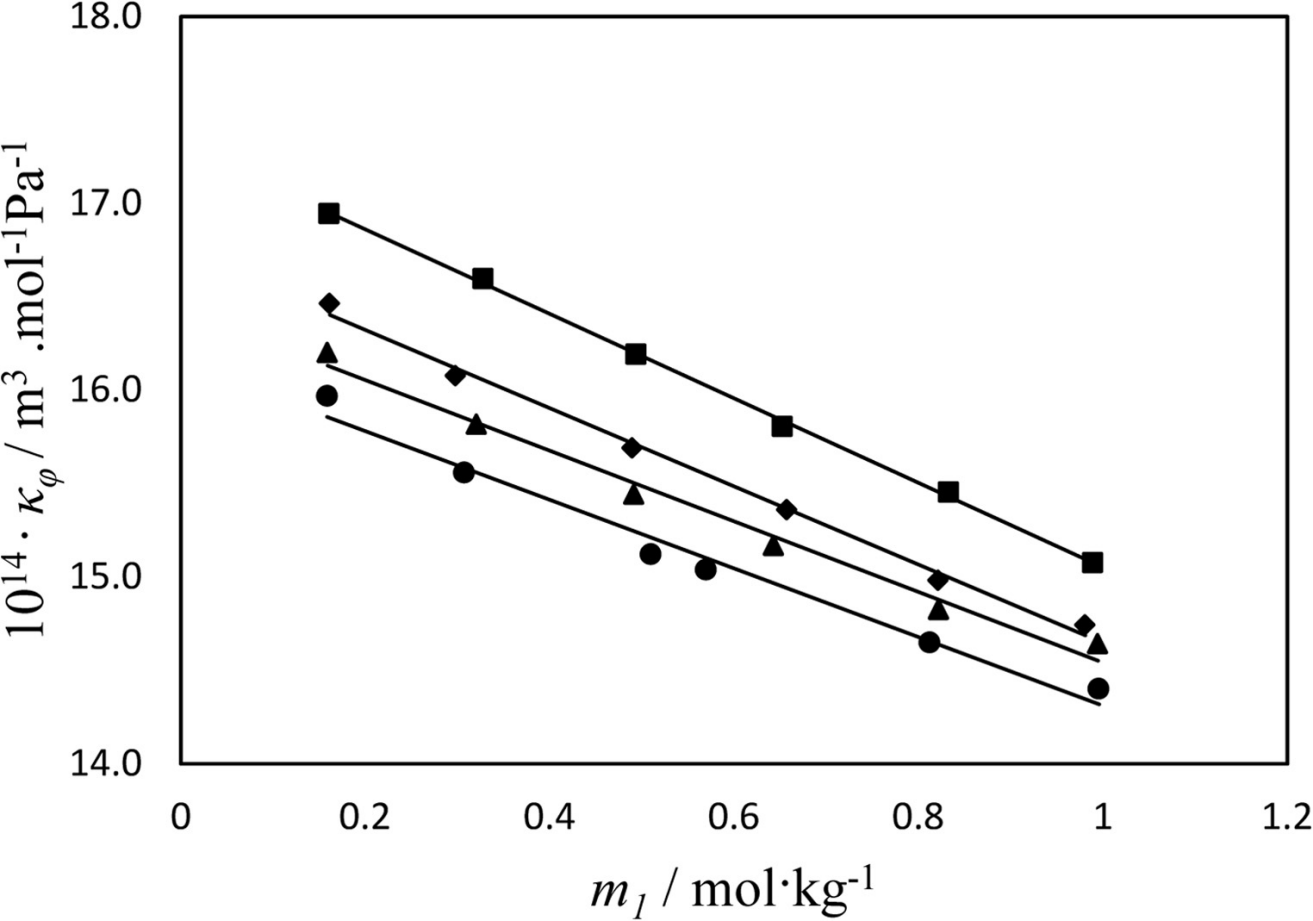
Apparent molar isentropic compressibility of D-calcium pantothenate, ${K_\varphi }$, versus its molality, *m*_1_, in aqueous ChCl/G solutions at *T* = 298.15 K: ●, binary; ▲, 0.1; ♦, 0.2; ■, 0.3 mol·kg^−1^.

The apparent molar isentropic compressibility, (${\kappa _\varphi }$) is obtained from the Eq. (8):

8 $${\kappa _\varphi }=\frac{{({\kappa _s}{d_0} - {\kappa _{s0}}d)}}{{md{d_0}}}+\frac{{{\kappa _s}M}}{d}$$

where ${\kappa _{s0}}$, and ${\kappa _s}$ are isentropic compressibility values of the solvent and solutions, respectively. The calculated ${\kappa _\varphi }$values with combined uncertainty of 0.02 × 10^−14^ m^3^∙mol∙Pa^−1^ for investigated systems are given in Table 2. Also, the apparent molar isentropic compressibility values for D-calcium pantothenate in the aqueous solutions of mixtures of ChCl/S, ChCl/G, or ChCl/F with concentration of 0.3 mol.kg^−1^ of each ChCl/S, ChCl/G, or ChCl/F at 298.15 K are represented in Fig. 2. As can be seen from Table 2, the values of apparent molar isentropic compressibility increase with increasing of temperature and concentration of studied mixtures of ChCl/S, ChCl/G, or ChCl/F. The variation of apparent molar isentropic compressibility with molality of D-calcium pantothenate can be presented by using the Eq. (9):

9 $${\kappa _\varphi }=\kappa _{\varphi }^{0}+{S_k}{m^{1/2}}+{b_k}m$$

where $\kappa _{\varphi }^{0}$, is partial molar isentropic compressibility, ${S_k}$ and ${b_k}$ have the empirical and similar meaning as in Eq. 2 for apparent molar volumes. The values of $\kappa _{\varphi }^{0}$, ${S_k}$ and ${b_k}$ are given for the studied solutions along with standard deviations at the experimental temperatures in Table 6. The small ${b_k}$ values rather than $\kappa _{\varphi }^{0}$ predict that solute-solute interactions are negligible in comparison to solute–solvent interactions. The $\kappa _{\varphi }^{0}$ values increase from negative values towards positive values by increasing the ChCl/S, ChCl/G, or ChCl/F concentration and temperature. By increasing mixtures of ChCl/S, ChCl/G, or ChCl/F concentration the formation of ion-pairs cause to suppressed the electrostatic interactions between D-calcium pantothenate and water molecules which leads to become more compressible than that at lower concentration^45^. The $\kappa _{\varphi }^{0}$values for D-calcium pantothenate in aqueous solutions of different DESs have similar order with $V_{\varphi }^{0}$and confirm the volumetric results: ChCl/S,> ChCl/G > ChCl/F, respectively.

**Table 6:** Partial molar isentropic compressibility ($~K_{\varphi }^{0}~$), adjustable parameters of Eq. 18 ($~{S_k}~$and $~{B_k}~$), transfer isentropic compressibility ($~{\Delta _{tr}}K_{\varphi }^{0}~$), and standard deviations ($\sigma (~K_{\varphi }^{0}~)$) for D-calcium pantothenate in aqueous solutions of mixtures of ChCl/S, ChCl/G, or ChCl/F at different temperatures^*a*^.

T / K	$~K_{\varphi }^{0}~$⋅10^14^/ ( m^3^·mol^−1^·Pa^−1^)	$~{S_k}~$·10^14^ / ( m^3^·mol^−2^·Pa^−1^·kg )	$~{B_k}~$·10^14^ / ( m^3^·mol^−2^·Pa^−1^·kg )	$~{\Delta _{tr}}K_{\varphi }^{0}~$·10^14^/ ( m^3^·mol^−1^·Pa^−1^)	$\sigma (~K_{\varphi }^{0}~)$·10^14^/ ( m^3^·mol^−1^·Pa^−1^)
( ChCl/S molar ratio of 2.000:1.000) + D-calcium pantothenate + water					
*m*_2_ = 0.100 mol·kg^−1^					
288.15	17.077	-0.842	-1.824	0.808	0.035
298.15	17.386	-1.332	-1.393	0.939	0.047
308.15	17.560	-1.452	-1.313	0.939	0.015
318.15	17.209	0.315	-2.592	0.449	0.018
*m*_2_ = 0.200 mol·kg^−1^					
288.15	18.261	-2.404	-0.825	1.992	0.027
298.15	18.774	-3.127	-0.546	2.327	0.019
308.15	18.680	-2.672	-0.829	2.059	0.035
318.15	19.407	-3.599	-0.382	2.647	0.038
*m*_2_ = 0.300 mol·kg^−1^					
288.15	18.476	-0.479	-2.276	2.207	0.020
298.15	18.832	-0.751	-1.998	2.385	0.027
308.15	18.931	-0.789	-2.020	2.310	0.024
318.15	19.660	-1.640	-1.433	2.900	0.038
( ChCl/G molar ratio of 2.000:1.000) + D-calcium pantothenate + water					
*m*_2_ = 0.100 mol·kg^−1^					
288.15	16.643	-1.324	-0.936	0.374	0.047
298.15	17.13	-2.143	-0.381	0.683	0.028
308.15	17.42	-2.430	-0.277	0.799	0.049
318.15	17.079	-0.604	-1.586	0.319	0.021
*m*_2_ = 0.200 mol·kg^−1^					
288.15	17.309	-2.192	-0.552	1.04	0.024
298.15	17.208	-1.439	-1.074	0.761	0.025
308.15	17.544	-2.027	-0.627	0.923	0.013
318.15	17.286	-0.860	-1.318	0.526	0.007
*m*_2_ = 0.300 mol·kg^−1^					
288.15	17.665	-2.023	-0.722	1.396	0.032
298.15	17.337	-0.069	-2.213	0.890	0.021
308.15	17.899	-0.954	-1.722	1.278	0.025
318.15	17.876	-0.114	-2.333	1.116	0.023
( ChCl/F molar ratio of 2.000:1.000) + D-calcium pantothenate + water					
*m*_2_ = 0.100 mol·kg^−1^					
288.15	16.628	-1.302	-0.946	0.359	0.047
298.15	17.113	-2.113	-0.397	0.666	0.028
308.15	17.417	-2.43	-0.277	0.796	0.049
318.15	17.095	-0.77	-1.429	0.335	0.014
*m*_2_ = 0.200 mol·kg^−1^					
288.15	17.306	-2.193	-0.551	1.037	0.024
298.15	17.204	-1.440	-1.073	0.757	0.025
308.15	17.541	-2.028	-0.626	0.920	0.013
318.15	17.283	-0.861	-1.317	0.523	0.007
*m*_2_ = 0.300 mol·kg^−1^					
288.15	17.66	-2.022	-0.722	1.391	0.032
298.15	17.332	-0.069	-2.212	0.885	0.021
308.15	17.894	-0.954	-1.721	1.273	0.025
318.15	17.871	-0.114	-2.332	1.111	0.023

^*a*^The standard uncertainties (*u*) for temperature, pressure, molality of D-calcium pantothenate and DES are u(*T*) = 0.01 K; u(*p*) = 0.5 kPa; *u*(m) = 0.002; *u*(DES) = 0.004, respectively. The combined uncertainty for apparent molar isentropic compressibility is *u*_*c*_ (κ_ϕ_) = 0.02 × 10^−14^ m^3^mol Pa^−1^ (0.68 level of confidence).

^*b*^*m*_*2*_ is the molal concentration of DES in water.

Partial molar isentropic compressibility of transfer, ${\Delta _{tr}}\kappa _{\varphi }^{0}$, for water and the aqueous solutions of DESs have been calculated using the following equation:

10 $$\triangle_{tr}\kappa_\varphi^0=\kappa_\varphi^0\left(\mathrm{ChCl}/\mathrm S,\;\mathrm{ChCl}/\mathrm G,\;\mathrm{or}\;\mathrm{ChCl}/\mathrm F\;+\;\mathrm{water}\right)-k_\varphi^0\left(\mathrm{in}\;\mathrm{water}\right)$$

The values of ${\Delta _{tr}}\mathop \kappa \nolimits_{\varphi }^{0}$ for studied solutions are represented in Table 6. The values of $\kappa _{\varphi }^{0}$ for binary (D-calcium pantothenate + water) was taken from reference^38^. The sign of ${\Delta _{tra}}\mathop \kappa \nolimits_{\varphi }^{0}$ is positive in the studied systems and these values increase with a rise in the concentration of mixtures of ChCl/S, ChCl/G, or ChCl/F. Positive values of $\mathop \kappa \nolimits_{\varphi }^{0}$ for D-calcium pantothenate illustrates the dominance of the head charged groups N^+^ and COO^–^ of DESs with the ions of D-calcium pantothenate which increase with a rise in the concentration of mixtures of ChCl/S, ChCl/G, or ChCl/F. This behavior which observed for partial molar isentropic compressibility of transfer, are in good agreement with volumetric results and supports them.

### Molecular fingerprint and solvation behavior

The σ-profile is a crucial concept in COSMO-based thermodynamics, representing the charge distribution on a molecule’s surface. It acts as a unique fingerprint, indicating the likelihood of finding specific charge density values in segmented segments. COSMO models, such as COSMO-RS and COSMO-SAC, utilize σ-profiles to predict thermodynamic properties and molecule-environment interactions.

**Figure 3:**
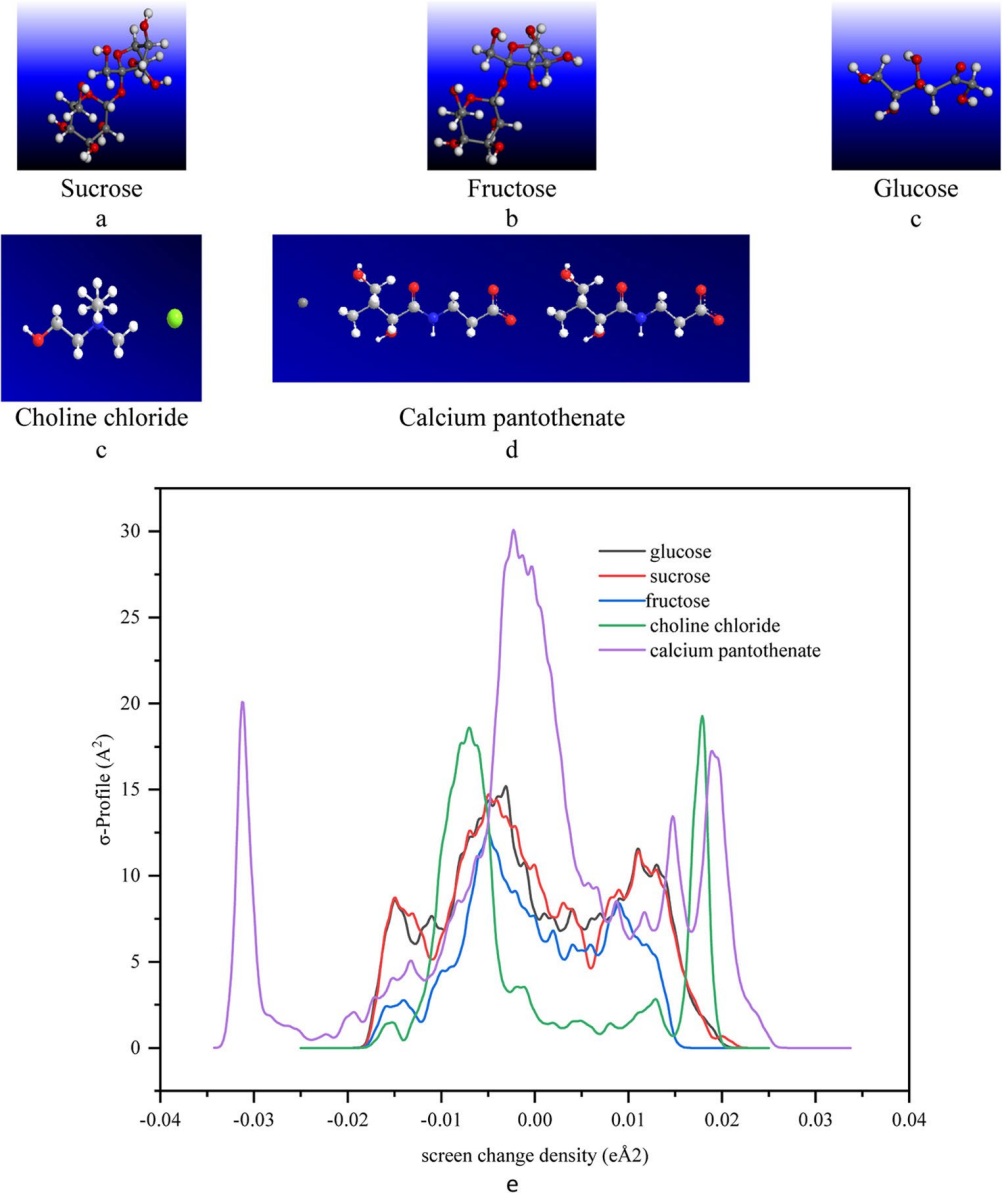
Optimized structure of (**a**) sucrose, (**b**) fructose, (**c**) glucose, (**d**) choline chloride, **d**) Calcium pantothenate, and (**e**) σ- profiles of these molecules.

These profiles are obtained through computational methods, primarily employing density functional theory (DFT) calculations, which can be computationally expensive. The utilization of the GGA VWN-BP function in Dmol3, as suggested by the developer. In this study, COSMO results were obtained through DFT calculations using the Dmol3 module of Materials Studio (Biovia, Materials Studio 2023). The molecule geometry was optimized using GGA (VWN-BP). Figure 3 presents the optimized structures of the molecules and the corresponding σ-profiles, for the studied molecules including choline chloride, glucose, fructose, sucrose, and calcium pantothenate. Additionally, Table 7 provides the cavity volume and cavity surface area results besides the HOMO and LUMO energy of the compounds from Dmol3 energy optimization calculations. These σ-profiles show that calcium pantothenate and choline chloride as ionic species show most negative and positive values due to the density of electron in the positive region with higher σ-profiles with higher intensity and vice versa for positive charge distribution in the negative region. The carbohydrates show negative and positive distributions around the 0 screen charge density. Choline chloride has the smallest cavity volume and surface area compared to the other molecules, suggesting a more compact solute. It also has the least negative dielectric (hydration) energy after calcium pantothenate, indicating a stronger interaction with the solvent compared to the other molecules. These results are compatible with the results are obtained in the previous sections. The results suggest that calcium pantothenate exhibits a different hydration behavior compared to the studied molecules (sucrose, fructose, glucose, and choline chloride). Smaller cavity volume and surface area, along with its less negative dielectric energy, indicate a more compact solute with weaker H-bonding interactions with the solvent. On the other hand, the studied molecules have larger cavity volumes and surface areas, stronger dielectric energies, and can form more intricate H-bond interactions with the solvent.

**Table 7:** The cavity volume and surface beside hydration energy, and highest occupied molecular orbital numbers and energy, lowest unoccupied molecular orbital of the studied compounds.

Compound	Cavity volume	Cavity surfacer	Dielectric (hydration) energy	*n*_HOMO_	HOMO	*n*_LUMO_	LUMO
	Å^3^	Å^2^	kcal/mol		eV		eV
glucose	327.344	304.443	-50.34	91	-5.860	92	0.710
sucrose	327.344	304.443	-50.34	33	-5.850	34	0.731
fructose	185.035	191.088	-19.74	48	-6.208	49	-2.209
Choline chloride	174.831	186.137	-56.03	38	-5.071	39	0.398
Calcium pantothenate	520.088	509.675	-352.30	127	-4.800	128	-1.482

## Conclusion

This research study focused on investigating the thermodynamic properties of D-calcium pantothenate (also known as vitamin B5), an essential micronutrient, in aqueous solutions of three DESs comprising choline chloride as the hydrogen bond acceptor (HBA) and sucrose, fructose, and glucose as the hydrogen bond donors (HBDs) (with molar ratio of 2:1) at concentrations of 0.1, 0.2, and 0.3 mol·kg^−1^ at different temperatures.

Through the analysis of volumetric and acoustic properties, strong interactions were observed between D-calcium pantothenate and the studied DESs, predominantly characterized by hydrophilic-hydrophilic interactions between the solute and solvent. The Helper’s constant, ${({\partial ^2}{V_\varphi }^{0}/\partial {T^2})_p}$, demonstrated the structure-making nature of D-calcium pantothenate in the aqueous solutions of the mentioned DESs. Overall, the findings indicate that the addition of DESs to aqueous solutions of D-calcium pantothenate leads to the release of water molecules from the solute’s hydration layer. As a result, the interactions between D-calcium pantothenate and DESs become more pronounced and strengthened. These insights contribute to a better understanding of the behavior and interactions of D-calcium pantothenate in aqueous ChCl/S, ChCl/G, or ChCl/F solutions, offering valuable implications for potential applications in various fields.

## Data Availability

All data generated or analysed during this study are included in this article.
